# Earthquake impacts on microcrustacean communities inhabiting groundwater-fed springs alter species-abundance distribution patterns

**DOI:** 10.1038/s41598-018-20011-1

**Published:** 2018-01-24

**Authors:** Simone Fattorini, Tiziana Di Lorenzo, Diana M. P. Galassi

**Affiliations:** 1 Department of Life, Health and Environmental Sciences, University of L’Aquila, L’Aquila, Italy; 2 cE3c–Centre for Ecology, Evolution and Environmental Changes/Azorean Biodiversity Group and University of Azores, Angra do Heroísmo, Portugal; 3 Institute of Ecosystem Study of the CNR (ISE-CNR), Sesto Fiorentino, Florence Italy

## Abstract

Earthquakes are important natural events, yet their impacts on animal communities are poorly known. Understanding earthquake impacts on groundwater communities is essential to assess their resilience and hence to perform conservation actions. We investigated how a 6.3 M_w_ earthquake that occurred in 2009 altered the community structure (diversity, evenness, dominance, species abundance distributions and beta-diversity) of microcrustaceans (Crustacea Copepoda) inhabiting springs fed by the Gran Sasso Aquifer (Central Italy). Sampling was done in low-discharge (1997), high-discharge (2005), and post-seismic (2012) hydrological years. Stygobites (obligate groundwater species) and non-stygobites (non-obligate groundwater species) showed different patterns. A high-water discharge in 2005 altered abundance patterns of non-stygobites. The earthquake re-established former abundance patterns. Stygobites were less affected by high-water discharge in 2005, and showed strong increases in diversity and evenness after the earthquake. This effect was due to the fact that the earthquake induced a strong population decline of previously dominant stygobites (especially of *Nitocrella pescei*) in the aquifer, and subsequently at the main spring outlets, thus allowing a more equitable species-abundance distribution. These results highlight the importance of considering species ecology to understand the effects of a significant earthquake event on animal communities.

## Introduction

Earth is a dynamic planet, whose surface is continuously re-shaped by extreme, sudden events, such as fires, floods, storms, volcanic eruptions, earthquakes, and tsunamis. These phenomena are considered “natural disasters” from the human perspective, because they injure people and produce economic damages. From the ecosystem’s perspective, they are forms of disturbance, defined as any discrete event in time and space that disrupts ecosystem, community, or population structure, and changes resources, substrate availability, or the physical environment^1,2^. As severe phenomena of disturbance, natural disasters may affect biodiversity by increasing mortality and altering habitat quality^3,4^.

Human activities have recently increased the severity and frequency of some types of extreme events (such as storms and wildfires)^5–8^, which may therefore represent sources of threats for biodiversity conservation. Even extreme events that are not influenced by human activities, such as volcanic eruptions and seismic events (with the exception of earthquakes on a small scale caused partially or completely by human activities^9–11^) are areas of concern, because they impact on an already threatened biodiversity^12^. In general, the study of ecosystem responses to major disturbance events may produce important ecological and resource management insights^13^ and there is increasing literature on the effects of fires^14,15^, floods^16,17^, hurricanes^18–20^, tornadoes^21,22^, volcanic eruptions^23–25^, and tsunamis^26,27^ on biodiversity. However, information on how animal communities respond to the disturbance of seismic events is still very limited^12,28^. In particular, the consequences of earthquakes on invertebrate biodiversity have been rarely addressed and remain therefore largely unknown^29–32^.

Although earthquakes can happen in any part of the world, the frequency of earthquakes is higher in the areas of boundaries between lithospheric plates. One of these seismically active areas is the Mediterranean-Alpine-Himalayas region, which extends from the Azores to the eastern coast of Asia^33^. Placed in the centre of the Mediterranean Basin, Italy is frequently hit by strong (6.0–6.9 M_w_) and sometimes major (7.0–7.9 M_w_) earthquakes (https://en.wikipedia.org/wiki/List_of_earthquakes_in_Italy). In the last 20 years, for example, seven strong earthquakes have occurred in Italy, among which the 6.3 M_w_ that struck the city of L’Aquila on 6 April 2009. This earthquake had a profound impact on the hydrogeological setting of the Gran Sasso Aquifer (GSA) by inducing an increase in the bulk hydraulic conductivity of the recharge area, near the ruptured fault zone together with fracture clearing and/or microcrack formations, and led to an anomalous rising of the water table (up to one metre) and flow rate (≥30% of the previous 15 years) in discharge zones^34^. As a result, the groundwater flow of the Tirino River valley, where ~65% of the aquifer discharge is located^35^, was altered, with important changes in the discharge of the Tirino Springs (TS), which are the main outlets of GSA, and which changed from rheo-limnocrene to predominantly limnocrene^36,37^. Previous research demonstrated that these changes altered the community organisation of subsurface (i.e. below the spring bed) microcrustaceans at TS, by reducing the abundance of obligate groundwater species (i.e. stygobites)^30^ and their spatial niche overlap^32^.

Understanding the impacts of earthquakes on groundwater communities is crucial to assess the resilience and sustainability of subterranean ecosystems and hence to perform conservation actions, such as a strict regulation of water extraction. In the present paper, we investigate if the earthquake altered the microcrustacean community structure in terms of diversity, evenness, dominance, species abundance distributions and beta-diversity. In general, disturbance events are expected to reduce diversity and evenness^1^, leading species abundance distributions to shift towards patterns characterized by a higher dominance of a few species^14,38–40^.

In two previous papers^30,32^, we demonstrated that L’Aquila earthquake has had profound impacts on microcrustacean population density, species spatial segregation and niche overlap. Using the same data set, in the present, complementary paper, we test if the earthquake decreased diversity and changed species abundance distributions by comparing microcrustacean communities in low-discharge (1997), high-discharge (2005), and post-seismic, very high-discharge (2012) hydrological years. As in the previous papers^30,32^, we used copepods (Crustacea Copepoda) because they comprise ~80% of the total abundance of meiofauna community at TS^30,36,37^, being therefore the best suited model organisms for this type of study.

## Results

### Comparison of pre-seismic communities

A total of 22 copepod species (9 stygobites and 13 non-stygobites) were found in both pre-seismic sampling years; and 18 copepod species in the post-seismic sampling year. The two pre-seismic communities showed similar values in most diversity indices (Table 1). When stygobites and non-stygobites were considered together, significant differences were only found for Simpson dominance (*P* = 0.022) and Berger–Parker dominance (*P* < 0.001) (Table 1). Simpson dominance was lower in 2005 than in 1997, which is a reflection of the increased relative abundance of certain species. In particular, the non-stygobiotic *Pesceus schmeili* (Mrázek, 1893), which accounted for 9.9% of the total copepod abundance in 1997, represented 24.4% of total copepod abundance in 2005; the non-stygobiotic *Moraria poppei meridionalis* Chappuis, 1929, which accounted for 0.6% of the total copepod abundance in 1997, was 16.8% in 2005; the non-stygobiotic *Bryocamptus typhlops* (Mrázek, 1893), which accounted for 4.8% of the total copepod abundance in 1997, represented 10.3% of the individuals in the 2005 community. Decrease in the Berger–Parker dominance is explained by the fact that this index is simply the proportion of the most abundant species. The most dominant species in the 1997 community was the stygobiotic *Nitocrella pescei* Galassi & De Laurentiis, 1997 with 33.0% of the individuals, followed by the non-stygobiotic *Bryocamptus echinatus* (Mrázek, 1893) with 10.4% of the individuals; no other species had a relative abundance >10%. By contrast, in the 2005 community, four species had abundance values >10%: *P. schmeili* (24.4%), *M. poppei meridionalis* (16.8%), *B. typhlops* (10.3%) and *N. pescei* (21.7%).

**Table 1:** Diversity, dominance and evenness indices calculated for the copepods of the Tirino Springs (Central Italy) in pre-seismic (1997 and 2005) and post-seismic (2012) years.

	1997	2005	2012	*P*	*P*	*P*
				(1997–2005)	(1997–2012)	(2005–2012)
**All species**						
Shannon diversity	2.151 (2.086–2.216)	2.112 (2.075–2.146)	2.300 (2.234–2.351)	0.275	<**0.001**	<**0.001**
Menhinick diversity	0.667 (0.667–0.667)	0.401 (0.401–0.401)	0.597 (0.564–0.597)	0.109	0.249	0.865
Margalef diversity	2.899 (2.899–2.899)	2.525 (2.525–2.525)	2.495 (2.348–2.495)	0.111	0.134	1.000
Simpson dominance	0.173 (0.158–0.188)	0.158 (0.153–0.165)	0.136 (0.126–0.149)	**0.022**	<**0.001**	<**0.001**
Berger-Parker dominance	0.330 (0.299–0.358)	0.244 (0.228–0.260)	0.250 (0.222–0.278)	<**0.001**	<**0.001**	0.697
Buzas-Gibson evenness	0.409 (0.384–0.437)	0.394 (0.379–0.408)	0.554 (0.520–0.590)	0.819	<**0.001**	**0.001**
Pielou evenness	0.706 (0.685–0.728)	0.694 (0.682–0.705)	0.796 (0.774–0.816)	0.690	<**0.001**	<**0.001**
**Non stygobites**						
Shannon diversity	1.923 (1.853–1.990)	1.682 (1.635–1.725)	1.893 (1.826–1.947)	<**0.001**	0.542	<**0.001**
Menhinick diversity	0.593 (0.593–0.593)	0.307 (0.307–0.307)	0.409 (0.409–0.409)	0.552	0.302	0.991
Margalef diversity	1.829 (1.829–1.829)	1.602 (1.602–1.602)	1.519 (1.519–1.519)	0.565	0.302	0.600
Simpson dominance	0.176 (0.162–0.192)	0.244 (0.233–0.256)	0.196 (0.181–0.213)	<**0.001**	0.091	<**0.001**
Berger-Parker dominance	0.252 (0.230–0.296)	0.375 (0.352–0.398)	0.314 (0.283–0.349)	<**0.001**	**0.028**	**0.003**
Buzas-Gibson evenness	0.570 (0.532–0.612)	0.414 (0.395–0.432)	0.604 (0.564–0.637)	**0.016**	0.790	<**0.001**
Pielou evenness	0.774 (0.746–0.802)	0.656 (0.636–0.672)	0.790 (0.761–0.812)	**0.001**	0.718	<**0.001**
**Stygobites**						
Shannon diversity	1.157 (1.075–1.236)	1.060 (1.002–1.116)	1.399 (1.280–1.495)	0.055	**0.004**	<**0.001**
Menhinick diversity	0.373 (0.373–0.373)	0.258 (0.258–0.258)	0.511 (0.438–0.511)	0.127	0.902	0.298
Margalef diversity	1.256 (1.256–1.256)	1.019 (1.019–1.019)	1.146 (0.955–1.146)	0.133	0.741	0.590
Simpson dominance	0.413 (0.384–0.444)	0.449 (0.422–0.479)	0.302 (0.265–0.351)	0.125	**0.001**	<**0.001**
Berger-Parker dominance	0.561 (0.520–0.600)	0.622 (0.591–0.652)	0.457 (0.383–0.527)	**0.019**	**0.015**	<**0.001**
Buzas-Gibson evenness	0.354 (0.326–0.383)	0.361 (0.341–0.385)	0.579 (0.521–0.653)	0.864	**0.004**	0.134
Pielou evenness	0.527 (0.490–0.563)	0.510 (0.482–0.538)	0.719 (0.665–0.773)	0.611	**0.001**	**0.023**

95% CI are given in parentheses. Probabilities refer to between-year comparisons for equal values based on 9999 permutations. Significant (*P* < 0.05) values are in bold.

### Comparison of pre- and post-seismic communities

The post-seismic community showed lower values of dominance and higher values of diversity and evenness in comparison with both pre-seismic communities. Namely, the post-seismic community differed significantly (*P* < 0.001) from the two pre-seismic communities for all indices reported in Table 1 except the Margalef and Menhinick indices (which only consider total richness and total abundance) and for the Berger–Parker index related to 2005–2012. The post-seismic community had a slightly lower richness (18 species) compared with that of the two pre-seismic communities (which had the same number of species: 21 species in both cases). Regarding total abundance (expressed as the number of individuals found in the total volume of sampled water, i.e. 1920 L each year), the post-seismic community had a total abundance (910 individuals, i.e. 0.47 individuals L^−1^) very similar to the 1997 pre-seismic community (992 individuals, i.e. 0.52 individuals L^−1^). The 2005 pre-seismic community included a larger number of sampled individuals (2750 individuals, i.e. 1.43 individuals L^−1^), i.e. the sampled total abundance was about three times higher, but this difference is strongly reduced in the index calculation, because the total abundance is logarithmised in the Margalef index and square-rooted in the Menhinick index. In 2012, three of the four most dominant species in 2005 returned to lower abundances (*P. schmeili*: 7.9%, *M. poppei meridionalis*: 4.1%, and *Nitocrella pescei*: 9.5%; *B. typhlops* increased to 20.1%).

### Comparison of pre- and post-seismic communities for non-stygobites

When stygobites and non-stygobites are analysed separately, different patterns emerge (Table 1). The non-stygobiotic species showed a significant (*P* < 0.001) reduction in diversity and evenness and an increase in dominance in the 2005 community compared with the 1997 community (Table 1). The post-seismic community differed from the 1997 community only for an increase in Berger-Parker dominance (*P* = 0.028), whereas it differed from the 2005 community for significant (*P* < 0.001) increases in diversity and evenness and reduction in dominance indices (Table 1).

### Comparison of pre- and post-seismic communities for stygobites

For the stygobites, the two pre-seismic years differed only for the Berger-Parker dominance (higher in 2005, *P* = 0.019), whereas the post-seismic community showed higher diversity (0.001 < *P* < 0.004) and evenness (0.001 < *P* < 0.023), and lower dominance (*P* < 0.001), in comparison with the two pre-seismic communities (Table 1).

### Species-abundance analysis

**Figure 1:**
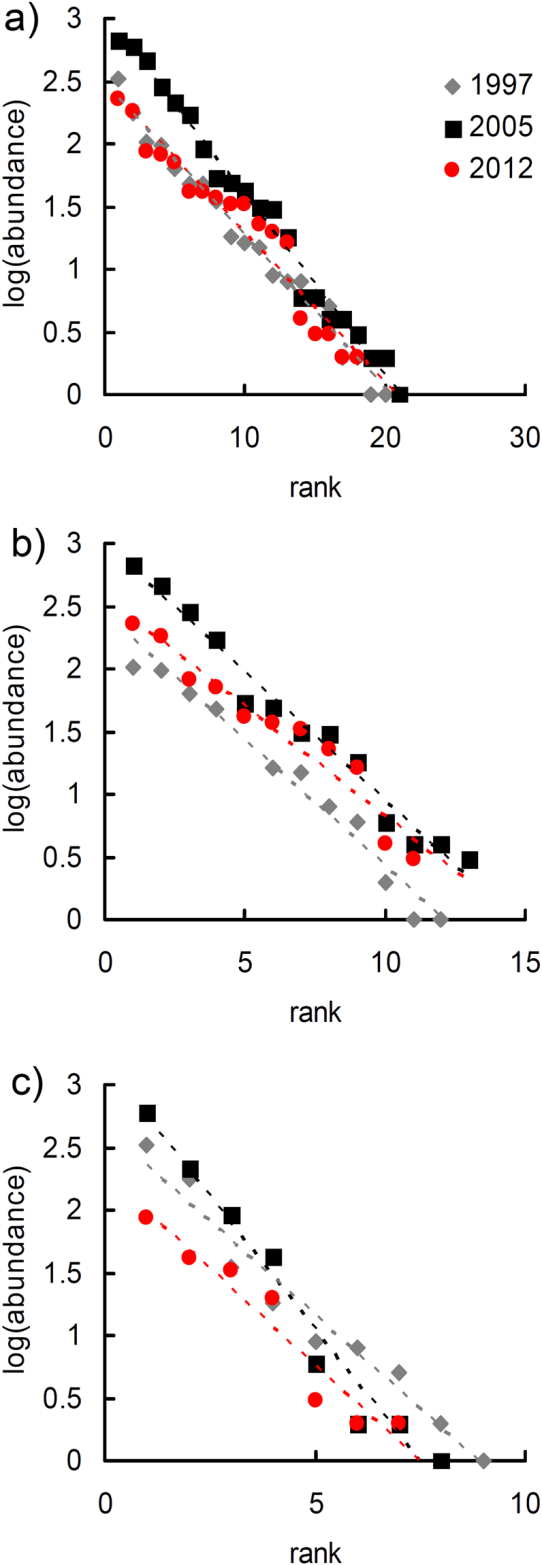
Rank-abundance distribution of the copepods of Tirino Springs (Central Italy) in pre-seismic (1997, grey diamonds; 2005, black squares) and post-seismic (2012, red dots) years. Panel a: comparison for all species. Regression statistics for the 1997 pre-seismic community: log(abundance) = (−0.122 ± 0.004) × rank + (2.479 ± 0.044), *R*² = 0.984, *F*_1,19_ = 1190.221, *P* < 0.0001. Regression statistics for the 2005 pre-seismic community: log(abundance) = (−0.144 ± 0.004) × rank + (3.026 ± 0.045), *R*² = 0.988, *F*_1,19_ = 1593.945, *P* < 0.0001. Regression statistics for the post-seismic community: log(abundance) = (−0.119 ± 0.007) × rank + (2.478 ± 0.081), *R*² = 0.941, *F*_1,17_ = 255.883, *P* < 0.0001. The 1997 community followed the lognormal series series but was modelled here for comparative purposes. Panel b: comparison for non-stygobites. Regression statistics for the 1997 pre-seismic community: log(abundance) = (−0.201 ± 0.013) × rank + (2.433 ± 0.094), *R*² = 0.961, *F*_1,10_ = 245.442, *P* < 0.0001. Regression statistics for the 2005 pre-seismic community: log(abundance) = (−0.205 ± 0.010) × rank + (2.998 ± 0.076), *R*² = 0.977, *F*_1,11_ = 461.579, *P* < 0.0001. Regression statistics for the post-seismic community: log(abundance) = (−0.175 ± 0.015) × rank + (2.573 ± 0.100), *R*² = 0.940, *F*_1,9_ = 141.585, *P* < 0.0001. Panel c: comparison for stygobites. Regression statistics for the 1997 pre-seismic community: log(abundance) = (−0.299 ± 0.023) × rank + (2.654 ± 0.127), *R*² = 0.961, *F*_1,8_ = 174.495, *P* < 0.0001. Regression statistics for the 2005 pre-seismic community: log(abundance) = (−0.422 ± 0.033) × rank + (3.161 ± 0.165), *R*² = 0.965, *F*_1,6_ = 166.370, *P* < 0.0001. Regression statistics for the post-seismic community: log(abundance) = (−0.307 ± 0.039) × rank + (2.292 ± 0.173), *R*² = 0.926, *F*_1,5_ = 62.997, *P* = 0.0005. In all cases, errors refer to standard errors.

The copepod community before the earthquake was best fitted by a lognormal model in 1997 and by a geometric series in 2005; in the post-seismic year (2012) the geometric series and the lognormal series fitted the copepod species abundance distribution equally well (Table 2). Thus, the copepod community shifted from the 1997 rather equitable species abundance distribution (lognormal) to a distribution characterised by lower evenness (geometric series) in 2005, and finally returned to a more balanced distribution of species abundances in 2012, after the earthquake. Because the 2005 and the 2012 communities are adequately fitted by the geometric series, rank-abundance distributions were modelled using a regression approach (Fig. 1a). The slope of the 2012 line was significantly lower than the slope of the 2005 line (equality of slopes: *F* = 9.881, *P* = 0.003), which indicates that the post-seismic community was less influenced by the most dominant species.

**Table 2:** Comparison of rank-abundance models for the copepods of the Tirino Springs (Central Italy) in pre-seismic (1997 and 2005) and post-seismic (2012) years.

	All species			Non-stygobites			Stygobites		
	1997	2005	2012	1997	2005	2012	1997	2005	2012
**Broken-stick**									
AIC	393.699	1126.730	173.704	105.028	693.355	105.717	373.000	559.729	54.565
**Geometric series**									
α	0.265	0.277	0.221	0.304	0.393	0.311	0.574	0.621	0.462
AIC	137.541	159.734	138.879	78.904	110.896	84.179	74.119	50.571	42.096
**Lognormal**									
μ	2.811	3.866	3.292	2.9731	3.893	3.637	2.749	3.320	2.677
σ	1.554	1.526	1.197	1.147	1.592	1.145	2.100	2.182	1.358
AIC	127.040	540.864	139.517	115.039	253.429	94.716	96.358	79.513	48.091
**Zipf**									
p_1_	0.390	0.375	0.316	0.359	0.479	0.386	0.642	0.676	0.511
γ	−1.317	−1.276	−1.085	−1.113	−1.486	−1.170	−1.970	−2.088	−1.430
AIC	207.829	901.648	212.936	163.560	406.837	139.840	125.382	139.116	61.877

When stygobites and non-stygobites are analysed separately, the geometric series gave the best fit in all cases (Table 2). When rank-abundance distributions were modelled using a regression approach, no significant differences between slopes were found (1997 *vs*. 2005: *F* = 9.081, *P* = 0.779; 1997 *vs*. 2012: *F* = 3.146, *P* = 0.091; 2005 *vs*. 2012: *F* = 1.736, *P* = 0.203), which indicates that non-stygobiotic species showed no changes in the dominance pattern (Fig. 1b). For the stygobiotic species (Fig. 1c), slopes were significantly different between the two pre-seismic communities (*F* = 10.010, *P* = 0.007) and marginally different between the 2005 and the post-seismic community (*F* = 5.149, *P* = 0.044), but not between the 1997 and the post-seismic community (*F* = 0.030, *P* = 0.865), which indicates that the dominance of the stygobiotic species after the earthquake returned to be similar to the 1997 situation after the increase in 2005.

### Beta-diversity analysis

Average beta-diversity values were similar among years for the whole community (arithmetic mean ± SE: 0.419 ± 0.059 for 1997; 0.467 ± 0.070 for 2005; 0.444 ± 0.043 for 2012) and for the stygobiotic species (0.526 ± 0.065 for 1997; 0.632 ± 0.072 for 2005; 0.633 ± 0.049 for 2012; difference between 1997 and 2012 is in fact marginally significant), whereas increased significantly after the earthquake for the non-stygobites (0.340 ± 0.048 for 1997; 0.323 ± 0.044 for 2005; 0.535 ± 0.043 for 2012) (Table 3). Beta-diversity values observed in 1997 were significantly correlated with those recorded in 2005 for the entire community and for the non-stygobiotic species, but not for the stygobites (Table 3). Beta-diversity values observed in 1997 were significantly correlated with those recorded in 2012 only for the stygobites and no correlation was detected between 2005 and 2012 beta-diversity patterns (Table 3).

**Table 3:** Student t-tests and Mantel tests (Pearson correlation) for inter-spring beta-diversity (Morisita index) values.

Sampling year	Student tests			Mantel tests		
	All species	Non-stygobites	Stygobites	All species	Non-stygobites	Stygobites
1997 vs. 2005	*t* = −0.695 *P* = 0.493	*t* = 0.379 *P* = 0.708	*t* = −1.166 *P* = 0.254	*r* = 0.430 ***P* = 0.036**	*r* = 0.530 ***P* = 0.005**	*r* = 0.129 *P* = 0.560
1997 vs. 2012	*t* = −0.361 *P* = 0.721	*t* = −3.010 ***P* = 0.006**	*t* = −2.067 ***P* = 0.048**	*r* = 0.098 *P* = 0.625	*r* = −0.014 *P* = 0.951	*r* = 0.628 ***P* = 0.020**
2005 vs. 2012	*t* = 0.319 *P* = 0.752	*t* = −3.241 ***P* = 0.003**	*t* = −0.011 *P* = 0.991	*r* = 0.240 *P* = 0.205	*r* = −0.142 *P* = 0.566	*r* = 0.359 *P* = 0.071

Significant (*P* < 0.05) values are in bold.

## Discussion

Data about groundwater communities of unconsolidated (porous) or fractured aquifers are usually gathered by sampling drilling wells because they are directly connected with the aquifer^41–44^. However, groundwater-fed springs may represent a more interesting, albeit more complex, source of information in the case of karstic aquifers, because the structure of their animal communities is influenced by the full variety of habitats that occur across the entire aquifer and transport history^45^. Groundwater-fed springs host species belonging to different ecological categories, including crenic species (which dwell exclusively in the spring^46–48^ but that are rare among copepods), stygobiotic species (which colonize the spring from the aquifer^49^) and non-stygobiotic species (species that live in spring habitats being cold stenothermic or generalists, species coming from downstream, and species flushed out from surface waters of the recharge area of the aquifer^30,50,51^). The same disturbance event may affect non-stygobiotic and stygobiotic species in different ways. The high discharge that occurred in 2005 increased the dominance (and hence decreased diversity and evenness) of non-stygobiotic species (but not of the stygobiotic ones). The earthquake that occurred in 2009 hit this already perturbed community, by inducing a significant decrease in dominance, and a significant increase in diversity and evenness of non-stygobites, which led this group of species to return to the 1997 values. Therefore, the effect of the earthquake on non-stygobiotic species was to re-equilibrate a situation altered by the high discharge in 2005. This pattern can be explained by the colonisation dynamics of these species within the TS system. Non-stygobiotic species occurring in benthic habitats of the TS system (which is an aquitard, with the carbonate bedrock with several fractures and strong upwelling in the sediment matrix overlying the bedrock^30^) may reach the spring both via surface-water dispersal from downstream and via the aquifer when drifting from the surface recharge area^52,53^. Therefore spring colonisation by non-stygobites is not strictly dependent on groundwater dynamics^52^ but is primarily regulated by the sediment texture of the spring-bed characteristics^49^. Thus, the earthquake might have induced variations in diversity, evenness and dominance of non-stygobites by re-shaping the sediment texture of TS through the effect of an increased discharge, more than by a direct effect of the aquifer dynamics. However, re-arrangements of sediment texture could have been equally produced by the high discharge that occurred in 2005. Thus, the non-stygobiotic species were affected by both the 2005 anomalous discharge and the mainshock-induced high discharge. By contrast, stygobiotic species, being only affected by changes in the karst groundwater flow that is focused to spring outlets, were much more sensitive to the effects of the earthquake than to the anomalous 2005 discharge. In fact, the 2005 anomalous discharge did not change diversity, dominance and evenness of stygobites, probably because it was within the range of the hydrological changes to which these groundwater-dweller species are used to. Post-seismic values in diversity and evenness of stygobites were higher not only in comparison with the values recorded in 2005 (and characterised by a high dominance effect), but also in comparison with 1997 values. Differently from non-stygobiotic species, stygobites reflected more directly the ecological processes that occurred into the aquifer feeding the spring system^49,52,53^. The 2009 mainshock markedly changed the Gran Sasso groundwater flow^34,54,55^ as well as water isotope and chemical composition^37^, and these changes were mirrored by a variation in the stygobiotic assemblage composition^30,32^ that was observed also in this study. In fact, the increase in stygobiotic diversity recorded in 2012 occurred as a result of a reduction in the abundance of most species^30^ and was associated with an increase in niche overlap due to the redistribution of animals caused by the earthquake-triggered discharge^32^.

This scenario is paralleled by variations in species abundance distribution patterns. For the whole community, the lognormal series was identified as the best fit model in the 1997 community, whereas the 2005 community followed the geometric series, and the 2012 community was equally best fitted by both models. The geometric model predicts very uneven abundances, broken stick predicts very even abundances, while lognormal is intermediate and assumes a small number of very rare species^56^. Low productivity systems were claimed to have uneven species abundance distributions and be well fitted by a geometric series, while high productivity systems are well fitted by lognormal curves and exhibit the highest evenness^57^. Species abundance distributions was observed to change along a successional gradient in deciduous forest plots in Illinois, USA, with more lognormal, more even communities occurring late in succession just as for productivity, whereas early stages of succession (e.g. following the felling of trees for timber) followed a geometric series^58^. Since these pioneer observations, lognormal shapes have mainly been associated with “fully censused” communities^59^ regulated by a large number of biotic and abiotic factors that together produce a lognormal abundance distribution according to the central limit theorem of statistics^40,60–62^.

In the copepods of TS, the 2005 above-average discharge conditions induced a shift in the species abundance distribution from relatively even abundances (expressed by the lognormal model) to uneven abundances (expressed by the geometric series). The effect of the earthquake was to level the strongest differences in species abundances and hence to re-establish a species abundance distribution similar to that recorded in 1997, especially to the detriment of stygobiotic species. In other words, the earthquake decimated the whole community, but the impact was particularly severe on the species-abundance distribution of stygobites, hence allowing an increase in the diversity and evenness of the non-stygobiotic species, thus re-establishing a more balanced species abundance distribution in the whole community. Non-stygobiotic species showed similar patterns of abundance distribution in all three years, whereas stygobiotic species showed a significant increase in the slope of the geometric series between 1997 and 2005 and then a reduction after the earthquake. Thus, changes in the species abundance distribution were mostly driven by the stygobiotic component.

Stygobites and non-stygobites showed important differences also for changes in beta-diversity patterns between years. For the whole community and for the non-stygobiotic species, beta-diversity patterns remained similar between the two pre-seismic years, but changed between pre-seismic and post-seismic years. Thus, non-stygobites were not affected by the higher discharge in 2005, but the earthquake disrupted the previous patterns by increasing beta-diversity. In contrast, for the stygobiotic species, beta-diversity pattern of 2005 was different from those of 1997 and 2012, whereas 2012 beta-diversity pattern was similar to that of 1997. Thus, the effect of the earthquake for the stygobites was that of reconstructing a beta-diversity pattern similar to that observed in 1997. It is counter-intuitive that the earthquake determined an increase in diversity and evenness. However, this unexpected “positive” effect can be explained in consideration of the type of changes in the community structure determined by the seismic event. The earthquake changed species composition (three species disappeared)^30^ and lowered species abundances that were increased by the 2005 high discharge; this induced a strong population decline of the species that dominated copepod communities, thus allowing a more equitable species-abundance distribution, and hence higher diversity and evenness.

Evidence that natural disasters may severely threaten biodiversity typically refer to population decline due to destruction of resources^63,64^ whereas effects on communities may be much more complex and, in certain ecosystems, periodic disaster events may be necessary for maintaining or introducing variability in community structure^65–67^.

For example, communities of small mammals inhabiting areas hit by earthquakes, fires, clearcutting, and floods have low relative abundances and high species diversity^68–71^. Also, it has been observed that a reduction in food availability immediately after the disturbance might be a reason for low relative abundances^69,72^. In the case of spring copepods, it is difficult to invoke a reduction in food availability as a main reason for population decline, because particulate organic matter (POM), which constitutes a consistent food supply for copepods along with bacteria^73^, increased with the earthquake^30^. Rather, decline in species abundances after the earthquake was mainly due to a strong increase in hydraulic conductivity and to the consequent aquifer dewatering, which massively flushed out individuals of fracture-dwelling species^30,32^. Moreover, one of the disruptive consequences of the earthquake was that of redistributing the pre-seismic stygobiotic species and causing new co-occurrence patterns and interspecific interactions^32^. Although the post-seismic community showed structural parameters similar to those of the 1997, species abundances at the level of individual spring of the TS system and their distribution across springs were altered by the earthquake more than by the increased discharge in 2005.

The community dynamics in disturbed areas is highly dependent on the re-colonisation processes from source populations^25,74,75^. However, copepod re-colonisation did not occur at the TS system for the duration of our study. Changes in the aquifer structure due to the earthquake led to a change in the pre-seismic species patterns. These patterns could not be re-established by colonisation from source pools for two reasons. The first reason is that most stygobites present in the storage subsystems of the aquifer were flushed out during the mainshock; this led to a strong reduction of the populations living in the primary habitat, thus preventing subsequent recolonisation of the springs^32^. The second reason is related to the fact that discharge remained above average throughout 2012. During a high discharge event, a karst aquifer feeding a spring works like a hydraulic system under pressure^76^. The pressure in the main drain pushes the groundwater into the less permeable parts of the aquifer, thus producing what is called a “piston effect”, which consists in the propagation of the hydraulic pressure at large distances^76^. In this aquifer type, which includes large conductive systems with high flow rate and current velocity, most copepods live in the small fractures of annex capacitive subsystems, and, even if good swimmers, they remain somewhat confined to this habitat^30,32^.

Because of this piston effect, stygobiotic copepods that survived fracture cleaning during the dewatering phase were transported to, and remained trapped in, the less permeable part of the saturated zone^30,77,78^. Thus, the 2012 post-seismic community was only marginally influenced (if any) by recolonisation processes, and changes in its structure can be substantially attributed only to the effects of the earthquake.

## Conclusions

Contrary to expectations, the mainshock of L’Aquila earthquake on 6 April 2009 did not impact negatively on structural parameters of the copepod community, but re-established a more balanced species abundance distribution after the changes induced by the anomalous discharge occurred in 2005. This apparently paradoxical situation is a consequence of the different processes that characterised the stygobiotic and non-stygobiotic species, and highlights the importance of considering species ecology to understand the effects of a catastrophic event, especially when it hits a community comprised of species that differ markedly in their response to environmental changes. However, sorting groundwater taxa into ecological categories is not an easy task and involves a remarkable taxonomic effort, which complicates the study of groundwater ecosystems, when compared with surface-water ecosystems. For example, although identification to species may be important to study the response of Ephemeroptera-Plecoptera-Tricoptera (EPT) to floods^79^, it seems that, in general, identification to the genus level may be sufficient for defining ecological categories in most surface-water invertebrates^80^. By contrast, in the groundwater fauna, the same genus may include both stygobiotic and non-stygobiotic species, which have completely different adaptations, trophic roles and colonisation dynamics^81^. We are aware that ecological studies requiring taxonomic identifications to the species level are onerous, time consuming and can be performed only by trained people; yet our study demonstrates that it is necessary not to jump to misleading conclusions.

Groundwater communities are well known for their low resilience^82^ and disturbance events that negatively affect their populations may easily lead to local extinction^30,82^. Most groundwater species are phylogenetic and/or distributional relicts, thus they are species of high conservation concern^83^. Groundwater habitats are generally considered stable environments, but our study demonstrates that, in fact, they can be suddenly modified by natural changes and that copepod communities can be subject to profound alterations due to occasional, but strong disturbance events represented by earthquakes. Groundwater environments are under a variety of severe anthropogenic pressures, such as pollution and water extraction^43^. Thus, anthropogenic disturbances occurring in a community already stressed by an earthquake might have extremely negative consequences. Of course, we cannot avoid earthquakes, but we should address any effort to avoid, or at least to reduce, the impact of anthropogenic stressors.

## Materials and Methods

### Study area and sampling procedures

The TS area is a spring complex at the boundary of the Gran Sasso Aquifer (GSA) located in the Gran Sasso Massif in central Italy (Apennines mountain range), featuring the highest peak south of the Alps (Corno Grande, 2922 m a.s.l.) and characterised by a high- to moderate-altitude montane landscape with low human impact. The GSA is a karstic aquifer with fast-flowing sections (karstic conduits) and interconnected low-flowing water small chambers^30^. The TS is the largest GSA-fed spring system, receiving ~65% of the GSA discharge^35^. The short (~15 km) Tirino River originating from TS joins the Aterno-Pescara River before eventually emptying into the Adriatic Sea.

Mean annual discharge at TS was relatively low in the first sampling year (1997: mean ± SD: 5.68 ± 0.21 m^3^ s^−1^); it was above-average in the second sampling year (2005: 6.02 ± 0.26 m^3^ s^−1^) and was well above average in the third sampling year (2012: 7.14 ± 0.26 m^3^ s^−1^) due to a 3-yr rising in discharge caused by the 6.3-M_w_ 2009 earthquake, before slowly returning to pre-seismic discharge values in summer 2013^30^.

Copepods were collected at eight sampling sites at the TS system adopting a random sampling method with four temporal replicates of three samples of 20 L in each of the eight sampled sites, for a total of 96 samples (and hence 1920 L) in each year. Subsurface samples of water were collected from the springbed (sediment patches and karstic fractures) with a hand-made Bou-Rouch pump^32^ and mobile pipes hammered at each sampling site. For each replicate, a standardised sample size of 20 L was withdrawn, a volume of water/sediments that is sufficient to obtain reliable estimates of abundance of rare species^84^. The meiofauna was extracted by filtering the 20-L samples through a hand net (mesh size = 60 µm). Samples were preserved in 80% ethyl alcohol. Individuals were later counted and identified to species level. Species were assigned to two ecological categories: stygobites and non-stygobites. Stygobites (obligate groundwater species) are strictly dependent on groundwater to complete their life cycles, and are drifted or washed periodically to the aquifer outlets following the groundwater flow. Non-stygobites found in the springs are freshwater species that live on the springbed surface, or in sediment interstices (e.g., to avoid predation) or are habitat generalists. Some of them are drifted from surface waters of the recharge area of the aquifer, others can be defined as crenobionts, i.e. species that complete their life cycle in the stable and relatively cold thermal regime of surface spring waters; other are generalist species that can colonise the spring sediments from downstream via the surface hydrological continuum.

Further details about the study site, discharge patterns and sampling procedures are given elsewhere^30,36,37^. Primary data used in this study have been published in previous papers^30,32^.

### Statistical analysis

Because no single diversity index encompasses all the characteristics of an ideal index^85^, a combination of them that reflects richness, dominance, evenness, and relative abundance was used. Thus, the following community parameters were calculated to compare pre- and post-seismic communities:

1 $${\rm{Shannon}}\,{\rm{index}}({\rm{entropy}}):\,H\text{'}=-\sum \frac{{n}_{i}}{n}\,\mathrm{ln}(\frac{{n}_{i}}{n}),$$

where *n*_*i*_ is the abundance of species *i* and *n* is the overall abundance (total number of individuals); *H′* ranges from 0 (one species dominates the community completely) to high values for communities with many species, each with few individuals.

2 $${\rm{Simpson}}\,{\rm{dominance}}\,{\rm{index}}:\,D={\sum (\frac{{n}_{i}}{n})}^{2}.$$

D varies from 0 (all species are equally present) to 1 (one species dominates the community completely).

3 $${\rm{B}}{\rm{u}}{\rm{z}}{\rm{a}}{\rm{s}}\,-\,{\rm{G}}{\rm{i}}{\rm{b}}{\rm{s}}{\rm{o}}{\rm{n}}\,{\rm{e}}{\rm{v}}{\rm{e}}{\rm{n}}{\rm{n}}{\rm{e}}{\rm{s}}{\rm{s}}:E=\,{e}^{H^{\prime} /S},$$

where *H′* is the Shannon index and *S* is the total number of species. This index varies from 0 (highest dominance by a single species) to 1 (all species have the same abundance).

4 $${\rm{Margalef}}\,{\rm{index}}:\,{Mg}=({S}-1)/\mathrm{ln}({\rm{n}}).$$

5 $${\rm{Menhinick}}\,{\rm{index}}:\,{Me}={S}/\sqrt{{\rm{n}}}.$$

6 $${\rm{Pielou}}\,{\rm{equitability}}({\rm{evenness}}):\,{J}^{\prime} ={H}^{\prime} /{\rm{lnS}}.$$

7 $${\rm{Berger}}\mbox{--}{\rm{Parker}}\,{\rm{dominance}}:\,{d}={{n}}_{{\max }}/{n},$$

i.e. the number of individuals in the dominant species (*n*_*max*_) divided by *n*.

Properties of these indices are discussed elsewhere^40,86–88^.

Ninety-five percent confidence intervals for all these indices were computed with a bootstrap procedure with 9999 randomizations. To compare diversity indices of pre- and post-seismic communities, we generated 9999 random matrices with two columns (samples), each with the same row and column totals as in the original data matrix. The probability of obtaining the observed difference by random sampling from a unique parental population was calculated as the number of times that the absolute difference of the indices of a replicate pair exceeded or equalled that of the original samples. Calculations were done using PAST v. 3.0^89^.

We also investigated if the earthquake modified the species abundance distributions (SADs) because the study of SADs allows inferences about patterns in the commonness and rarity of species in a community beyond those that flow from many simple diversity indices and can therefore provide insights into the effects of disturbance on ecological communities^90^. We modelled SADs using rank-abundance curves^40,85^. In the abundance-rank representations, all the species in a community are ranked from the most to the least abundant. Each species has a rank, which is plotted on the horizontal axis, while its abundance is plotted on the vertical axis: the abundance for the most abundant species is plotted first, then the next most common and so on until the array is completed by the rarest species.

Several *a priori* established distributions can be used to model empirical rank-abundance curves^59^. We compared pre- and post-seismic SADs using a selection of widely applied SAD models that are appropriate for discrete distributions^56,91^: the geometric series, the broken stick model, the lognormal series, and the Zipf model.

In the geometric series, also known as the niche preemption model, each species takes a constant fraction (*α*) of the remaining resources and the expected abundance of a species at rank *r* is:

8 $${a}_{r}=J\alpha {(1-\alpha )}^{r-1}.$$

The only estimated parameter is the preemption coefficient *α*, which gives the decay rate of abundance per rank, whereas *J* is the total number of individuals.

The geometric series is the mathematical model used to express the niche preemption hypothesis, in which the sizes of the niche hypervolumes (measured by species relative abundances) are sequentially preempted by the most abundant to the least abundant species. If in the rank-abundance plot a log scale is used for abundance, the species exactly fall along a straight line, according to the equation:

9 $$\mathrm{log}({\rm{a}})={{b}}_{{0}}+{{b}}_{{1}}{r},$$

where *a* is the species abundance, *r* is the respective rank, and *b*_0_ (the intercept) and *b*_1_ (the slope) are optimised fitting parameters^39^. With this approach, it is possible to use the regression slope to compare different species assemblages that follow the same rank-abundance distribution^39^. Among all proposed SAD models, the geometric series represents the least equitable distribution and it is known to provide a good fit to simple communities characterised by the high dominance of a few species^40,85,92^. On the opposite, most equitable empirical distributions should be modelled by the broken stick (BS) model^93^. The BS model is theoretically questionable and communities rarely are correctly characterised by such model^88,94^. Yet, the BS model is useful in comparative analyses because it represents a simple benchmark in opposition to the geometric series.

In the broken stick model, the expected abundance of species at rank *r* is:

10 $${a}_{r}=(J/S){\sum }_{x=r}^{S}(1/x),$$

where *J* is the total number of individuals and *S* is the total number of species in the community^95^. In the BS there are no fitted parameters.

Note that another species abundance distribution model widely used in community ecology for communities dominated by few species is the log-series^96–98^. However, the geometric series and the log-series abundance distributions are interrelated and are two representations of, essentially, the same underlying abundance distribution^97,99^.

The lognormal is one of the most commonly used models for describing SADs^100^. It has been derived as a null form of the distribution resulting from the central limit theorem^97^, and it is classified among the purely statistical models^56^, but can be the limit of population dynamics^101^, or niche partitioning^102,103^.

The lognormal model assumes that the logarithmic abundances are distributed normally:

11 $${{a}}_{{r}}=\exp [\,\mathrm{log}({\rm{\mu }})+\,\mathrm{log}({\sigma }{\Phi })],$$

where *µ* and *σ* are, respectively, the mean and standard deviation of the variable’s natural logarithm, and *Φ* is a normal deviate.

The Zipf distribution (which is a type of power law probability distribution based on branching processes^56^) is:

12 $${a}_{r}=J{p}_{{1}}{r}^{\gamma }$$

where *p*_1_ is the fitted proportion of the most abundant species, and *γ* is a decay coefficient.

Following current best practices in the study of species abundance distributions^98,104^, we used maximum likelihood estimation to fit models^105–107^ and likelihood-based model selection to compare models^108^. The lognormal and Zipf models were fitted using generalized linear models with logarithmic link function. The preemption model was fitted as a non-linear (quasi Newton) algorithm. Since species abundances were expressed as count data, we used the Poisson error. We used the Akaike Information Criterion (AIC) to compare the fits of the different models^98,104,108^. All models were fitted and compared using the R package ‘vegan’ version 2.4-3^109^.

For communities that followed the geometric series, we also used the regression approach described above and tested the equality of slope with an ANCOVA approach using R^110^. We conducted all the analyses for all species and for stygobites and non-stygobites separately for the whole TS system.

Finally, we investigated how beta-diversity varied between years. To express between-spring beta-diversity we used the Morisita index, which is suggested as the most appropriate for quantitative data^111^. We tested for differences in average beta-diversity values between years using paired *t*-tests. Then, we correlated matrices of between-spring beta-diversity values using Mantel tests (Pearson correlation coefficient, 10000 permutations) to assess if patterns were similar between years. Calculations were done with the R package ‘vegan’ version 2.4-3^109^.
